# Rheumatoid Arthritis Does Not Negatively Impact Outcomes of Patients Admitted for Atrial Fibrillation

**DOI:** 10.7759/cureus.10241

**Published:** 2020-09-04

**Authors:** Ehizogie Edigin, Emmanuel Akuna, Iriagbonse Asemota, Precious Eseaton, Pius E Ojemolon, Hafeez Shaka, Augustine Manadan

**Affiliations:** 1 Internal Medicine, John H. Stroger, Jr. Hospital of Cook County, Chicago, USA; 2 Internal Medicine, University of Benin, Benin City, NGA; 3 Anatomical Sciences, St. George's University, St. George, GRD; 4 Rheumatology, John H. Stroger, Jr. Hospital of Cook County, Chicago, USA

**Keywords:** rheumatoid arthritis, atrial fibrillation, conduction disorders, pharmacologic cardioversion, ablation

## Abstract

Objectives

This study aimed to compare the outcomes of patients primarily admitted for atrial fibrillation (AF) with and without a secondary diagnosis of rheumatoid arthritis (RA). The primary outcome of interest was inpatient mortality. Hospital length of stay (LOS), total hospital charges, and odds of undergoing ablation and pharmacologic cardioversion were the secondary outcomes of interest.

Methods

Data were abstracted from the National Inpatient Sample (NIS) 2016 and 2017 databases. The NIS is the largest hospitalization database in the United States (US). The NIS was searched for hospitalizations for adult patients with AF as principal diagnosis with and without RA as secondary diagnosis using the International Classification of Diseases, 10th Revision (ICD-10) codes. Multivariate logistic and linear regression analysis was used accordingly to adjust for confounders.

Results

There were over 71 million discharges in the combined 2016 and 2017 NIS database. Out of 821,630 AF hospitalizations, 17,020 (2.1%) had RA. Hospitalizations for AF with RA had 0.18 days' decrease in adjusted mean LOS (p=0.014), and lower total hospital charges ($38,432 vs $39,175, p=0.018) compared to those without RA. AF hospitalizations with RA had similar inpatient mortality [1.1% vs 0.91%, adjusted odds ratio (AOR): 0.90, 95% CI: 0.63-1.27, p=0.540] and odds of undergoing ablation (3.5% vs 4.2%, AOR: 1.1, 95% CI: 0.87-1.30, p=0.549) and pharmacologic cardioversion (0.38% vs 0.38%, AOR: 1.00, 95% CI: 0.53-1.89, p=0.988) compared to those without RA.

Conclusions

Patients admitted for AF with coexisting RA were found to have lesser adjusted mean LOS and lower total hospital charges compared to those without RA. However, inpatient mortality and the odds of undergoing ablation and pharmacologic cardioversion were similar between both groups.

## Introduction

Atrial fibrillation (AF) is the most clinically relevant rhythm disorder; it is characterized by irregularly irregular rhythm and is a common cause of cardiovascular (CV) morbidity and mortality [1,2]. Rheumatoid arthritis (RA) is a chronic autoimmune disease that causes inflammatory arthritis and is characterized by synovial joint involvement as well as significant extra-articular manifestations and complications [3,4].

There is an established association between RA and CV diseases. CV mortality and morbidity are significantly higher in RA patients, with more than half of all RA-related deaths attributable to CV diseases [4,5]. RA has been shown to increase the incidence of AF with sustained inflammation as the major driver [4,6]. In a meta-analysis of three prospective studies, the pooled risk ratio for the development of AF in RA patients was found to be 1.29 (95% CI: 1.05-1.59) compared to controls [4].

There is a scarcity of national population-based research on the outcomes of AF hospitalizations in RA patients compared to non-RA patients. We sought to bridge this knowledge gap. Our study sought to compare the outcomes of AF hospitalization in patients with RA to those without RA using national-level population data. Additionally, our study also investigated the likelihood of undergoing ablation or pharmacologic cardioversion in patients with AF with RA compared to those without RA. We used the data for the two most recent years from the National Inpatient Sample (NIS) database to address these clinically important issues.

## Materials and methods

Data source

We conducted a retrospective study of hospitalizations with a principal diagnosis of AF with and without a secondary diagnosis of RA from the NIS 2016 and 2017 databases. The NIS is maintained by the Agency for Healthcare Research and Quality. It is the largest available all-payer inpatient public database in the United States (US). It is designed as a stratified probability sample to be representative of all acute-care, nonfederal hospitals in the US. Hospitals are stratified according to bed size, teaching status, ownership, geographic region, and urban/rural status. A 20% probability sample of all hospitals within each stratum is then obtained. All hospitalizations from these hospitals are recorded and then weighted to ensure they remain representative nationally. Data from 47 statewide data organizations are included in the NIS 2016 and 2017 sampling frame. As much as 30 discharge diagnoses for each hospitalization can be recorded using the International Classification of Diseases, Tenth Revision (ICD-10) codes in NIS 2016, and 40 discharge diagnoses in the NIS 2017 database. Diagnoses are divided into principal and secondary diagnoses in the NIS database. The principal diagnosis is the main reason for the hospitalization. Secondary diagnoses are any diagnoses other than the principal diagnosis. Secondary diagnoses that preceded the index admission cannot be reliably distinguished from those that started during the index admission. All patient information in the NIS are de-personalized and available publicly, hence institutional board review and approval was not obtained for our study. The description of the NIS database is similar to a previously published NIS paper [7].

Inclusion criteria and study variables

The study population consisted of all inpatient hospitalizations recorded in the NIS 2016 and 2017. Race, gender, age, hospital characteristics, medical comorbidities, and primary and secondary outcomes were the study variables of interest. We used the following ICD-10 codes to identify principal/secondary diagnoses: AF - all 148.0, 148.1, 148.2, 148.91 codes; and RA - all M05 and M06 codes [see the supplementary table (Table 4) in the Appendix]. We studied baseline characteristics and outcomes for AF hospitalization with and without RA.

Outcomes

The primary outcome of interest was inpatient mortality. Hospital length of stay (LOS), total hospital charge, and odds of undergoing ablation and pharmacologic cardioversion were the secondary outcomes of interest. AF Hospitalizations with ablation were obtained by using ICD procedure codes (all 0258 codes) for "destruction of the conduction system of the heart", while those with pharmacologic cardioversion were obtained using ICD-10 procedure codes for "parenteral administration of antiarrhythmic agents".

Statistical analysis

Statistical analyses were performed using STATA, version 16 (StataCorp, College Station, TX). A univariate logistic regression analysis using all variables and comorbidities in Table 1 was used to calculate unadjusted odds ratios (ORs) for the primary outcome. All variables with p-values of <0.1 were included in a multivariate logistic regression model. Univariate association of variables and comorbidities with the primary outcome, highlighting the variables included in the multivariable logistic regression model, are displayed in Table 2. P-values of <0.05 were considered significant in the multivariate analysis. Confounders were selected from the literature review. The Charlson Comorbidity Index was used to adjust for comorbidity burden. A multivariate logistic and linear regression model with all variables and comorbidities in Table 1 was used accordingly to adjust for confounders for the secondary outcomes.

## Results

There were over 71 million discharges included in the combined 2016 and 2017 NIS database. Of those, 821,630 hospitalizations were adult patients, who had a principal ICD-10 code for AF; 17,020 (2.1%) of these hospitalizations had RA as a secondary diagnosis, while 804,610 (97.9%) hospitalizations did not have RA as a secondary diagnosis. Characteristics of AF hospitalizations with and without coexisting RA are displayed in Table 1.

**Table 1:** Baseline characteristics of atrial fibrillation hospitalizations with and without rheumatoid arthritis *Median household income for the patient’s Zip Code AF: atrial fibrillation; RA: rheumatoid arthritis; MI: myocardial infarction; PCI: percutaneous coronary intervention; CABG: coronary artery bypass graft; COPD: chronic obstructive pulmonary disease; DM: diabetes mellitus; CHF: chronic congestive heart failure; CKD: chronic kidney disease; O_2_: oxygen; AICD: presence of automated implantable cardioverter defibrillator

Baseline characteristics	AF (n=821,630)		
	Without RA (n=804,610)	With RA (n=17,020)	P-value
Mean age in years	70.8	73.9	<0.0001
Female gender	51.00%	72.50%	<0.0001
Race			0.0121
White	82.20%	84.20%	Reference
Black	7.80%	7.40%	0.267
Hispanic	6.10%	5.40%	0.092
Asians	1.50%	0.94%	0.003
Native Americans	0.35%	0.39%	0.769
Others	2.10%	1.70%	0.09
Charlson Comorbidity Index			<0.0001
0	24.50%	0%	
1	26.30%	20.50%	
2	19.20%	25.90%	
≥3	30.00%	53.60%	
Hospital bed size			0.7351
Small	20.00%	19.90%	
Medium	30.50%	31.10%	
Large	49.60%	49.10%	
Hospital teaching status			0.1806
Nonteaching	38.00%	39.20%	
Teaching	62.00%	60.80%	
Hospital location			0.0661
Rural	11.10%	12.20%	
Urban	88.90%	87.80%	
Expected primary payer			<0.0001
Medicare	70.40%	82.70%	
Medicaid	6.20%	3.10%	
Private	21.00%	13.60%	
Self-pay	2.40%	0.69%	
Median household income* (quartile)			0.5206
1st (0-25th)	27.50%	26.80%	
2nd (26th-50th)	27.20%	28.10%	
3rd (51st-75th)	24.80%	25.10%	
4th (76th-100th)	20.60%	19.90%	
Hospital region			0.1116
Northeast	20.20%	19.00%	
Midwest	24.10%	25.70%	
South	40.70%	40.00%	
West	15.10%	15.20%	
Dyslipidemia	50.20%	51.40%	0.1786
Old MI	8.30%	10.00%	0.0006
Old PCI	1.10%	0.97%	0.4194
Old CABG	7.20%	7.00%	0.7158
Old pacemaker	5.40%	5.80%	0.3406
AICD	3.00%	2.00%	0.0012
COPD	19.40%	26.70%	<0.0001
Carotid artery disease	1.30%	1.70%	0.0821
Old stroke	0.62%	0.41%	0.1268
Hypertension	48.40%	48.50%	0.9559
Peripheral vessel disease	4.20%	5.10%	0.0053
Hypothyroidism	17.20%	24.40%	<0.0001
DM type 1 and 2	28.00%	24.60%	<0.0001
Obesity	19.60%	17.20%	0.0004
CHF	37.50%	41.80%	<0.0001
CKD	18.10%	20.10%	0.0026
Liver disease	3.00%	2.60%	0.1694
Electrolyte derangement	18.80%	22.00%	<0.0001
Maintenance hemodialysis	2.20%	1.50%	0.0098
O_2_ dependence	3.40%	5.00%	<0.0001
Smoking	27.60%	30.10%	0.0012
Anemia	15.70%	23.20%	<0.0001

Patients in the RA group were older (73.9 vs 70.8 years, p<0.0001) and the group had more females than males (72.5% vs 51.0%, p<0.0001). Also, the RA group had fewer cases of diabetes mellitus (24.6% vs 28%, p<0.0001) and obesity (17.2% vs 19.6%, p=0.0004). In contrast, the RA group had more smokers (30.1% vs 27.6%, p=0.0012) and more patients with peripheral vessel disease (5.1% vs 4.2%, p=0.0053) and old myocardial infarction (10% vs 8.3%, p=0.0006). The univariate association of variables and comorbidities with the primary outcome are displayed in Table 2.

**Table 2:** Univariate association of baseline variables with inpatient mortality *Variable included in the multivariable logistic regression model; **Median household income for the patient’s Zip Code MI: myocardial infarction; PCI: percutaneous coronary intervention; CABG: coronary artery bypass graft; COPD: chronic obstructive pulmonary disease; DM: diabetes mellitus; AICD: presence of automated implantable cardioverter defibrillator; CHF: chronic congestive heart failure; CKD: chronic kidney disease; O_2_: oxygen

Baseline variables	Odds ratio	P-value
Age*	1.06	<0.0001
Female gender*	1.19	<0.0001
Race*		
White	Reference	Reference
Black	1.17	0.085
Hispanic	1.11	0.325
Asians	1.19	0.421
Native Americans	0.99	0.979
Others	0.55	0.015
Charlson Comorbidity Index*	1.39	<0.0001
Hospital bed size		
Small	Reference	Reference
Medium	0.98	0.734
Large	0.91	0.167
Hospital teaching status	1.02	0.755
Hospital location	0.99	0.979
Expected primary payer*		
Medicare	Reference	Reference
Medicaid	0.58	<0.0001
Private	0.35	<0.0001
Self-pay	0.45	0.001
Median household income** (quartile)*		
1st (0-25th)	Reference	Reference
2nd (26th-50th)	0.93	0.293
3rd (51st-75th)	0.83	0.012
4th (76th-100th)	0.74	<0.0001
Hospital region*		
Northeast	Reference	Reference
Midwest	0.9	0.188
South	0.99	0.836
West	1.16	0.086
Dyslipidemia*	0.64	<0.0001
Old MI	0.88	0.228
Old PCI	0.83	0.49
Old CABG*	1.18	0.066
Old pacemaker	0.91	0.44
AICD	1.01	0.926
COPD*	2.05	<0.0001
Carotid artery disease	1.13	0.605
Old stroke*	7.61	<0.0001
Hypertension*	0.45	<0.0001
Peripheral vessel disease*	1.6	<0.0001
Hypothyroidism	1.01	0.912
DM type 1 and 2	1.03	0.583
Obesity*	0.49	<0.0001
CHF*	2.56	<0.0001
CKD*	2.43	<0.0001
Liver disease*	4.93	<0.0001
Electrolyte derangement*	4.82	<0.0001
Maintenance hemodialysis*	2.12	<0.0001
O_2_ dependence*	2.43	<0.0001
Smoking*	0.82	0.002
Anemia*	2.66	<0.0001

Of note, 7,520 adult AF hospitalization (0.9%) resulted in inpatient mortality; 190 (1.1%) of the deaths occurred amomg patients with coexisting RA vs 7,330 (0.9%) for those without coexisting RA (p=0.2121). Hospitalizations for AF with RA had 0.18 days' decrease in adjusted mean LOS (p=0.014), and lower total hospital charge ($38,432 vs $39,175, p=0.018) compared to those without RA. AF hospitalizations with RA had similar inpatient mortality [1.1% vs 0.91%, adjusted odds ratio (AOR): 0.90, 95% CI: 0.63-1.27, p=0.540] and odds of undergoing ablation (3.5% vs 4.2%, AOR: 1.1, 95% CI: 0.87-1.30, p=0.549) and pharmacologic cardioversion (0.38% vs 0.38%, AOR: 1.00, 95% CI: 0.53-1.89, p=0.988) compared to those without RA (Table 3).

**Table 3:** Clinical outcomes of atrial fibrillation hospitalizations with and without rheumatoid arthritis *Statistically significant LOS: length of stay at hospital; CI: confidence interval; AF: atrial fibrillation; RA: rheumatoid arthritis; USD: United States Dollar

Outcomes	AF with RA (n=17,020)	AF without RA (n=804,610)	Adjusted odds ratio	P-value
	%	%	(95% CI)	
Primary outcome				
In-hospital mortality	1.1	0.91	0.90 (0.63-1.27)	0.540
Secondary outcomes				
Ablation	3.5	4.2	1.1 (0.87-1.30)	0.549
Pharmacologic cardioversion	0.38	0.38	1.00 (0.53-1.89)	0.988
			Adjusted mean difference	
LOS, mean, days	3.7	3.4	-0.18 (- {0.33-0.04})	0.014*
Total charge, mean, USD	38,432	39,175	-2,035 (- {3,725-346})	0.018*

## Discussion

The major findings of this study were as follows: 1) no difference in mortality between AF hospitalizations with and without RA; 2) patients hospitalized for AF with RA had lesser adjusted LOS and total hospital charges compared to those without RA; and 3) AF hospitalizations with and without RA had similar rates of interventions such as ablation and pharmacologic cardioversion.

RA is the most common inflammatory joint disease with a significant CV disease burden [2]. The most common arrhythmia among RA patients is AF, with a higher incidence rate compared to the general population [2,8]. The underlying mechanism of increased risk of AF in patients with RA has been extensively studied and several explanations have been offered. Most studies point to the chronic inflammatory state with significant inflammatory marker levels playing an important role in the development of AF indirectly by accelerating the rate of ischemic heart disease and congestive heart failure and directly on the cardiac conduction system [9,5]. The direct effect proposed in another study on the conduction system includes the effect of elevated circulatory antibodies to the conducting system, which leads to increased P-wave dispersion and increase in left atrium (LA) size, which is a known predictor of AF [8]. A more recent study in 2020 by Milton Parker showed that increased epicardial adipose expansion and inflammation found in RA led to coronary microvascular dysfunction and fibrotic changes in the LA leading to myopathy, increased LA stiffness, and blood stasis, resulting in AF [10].

Traditional risk factors for CV and cerebrovascular diseases are generally more prevalent in RA patients [11]. The RA group in our study had fewer statistically significant traditional CV risk factors such as DM and obesity. However, the RA group had a statistically significant higher number of smokers and more patients with peripheral vascular disease, old myocardial infarction, chronic kidney disease, and congestive heart failure. RA also tends to alter the lipid profile, characterized by low high-density lipoprotein (HDL), and high pro‐atherogenic lipoprotein‐a (Lpa) levels [12,13]. Our study did not find any statistically significant difference in dyslipidemia between the RA and non-RA groups.

A significant number of studies have clearly shown the increased incidence risk of AF in RA patients. A cohort study using the Danish National Registry showed a 40% increased incidence rate in patients with RA compared to the general population [9]. In a meta-analysis, the pooled risk ratio for AF development was found to be 1.29, showing an increased risk for developing AF in RA patients [4].

However, very few studies have been conducted on the outcomes of AF in RA patients. A similar population-based cohort study in Olmsted County, Minnesota showed no difference in mortality between AF patients with and without RA [14]. The study was limited because it involved a small subset of the US population with only 1,626 patients. Our study used a nationwide US hospitalization database and it showed similar results to the study in Minnesota. Our study concluded that mortality outcome is not influenced by the presence of RA. More studies are needed to explore the mechanism behind our findings.

This study has several strengths. Firstly, it utilized information from the NIS, a large nationwide dataset, to provide a large sample size, thereby greatly increasing the power of the study. Secondly, the nature of the database allowed us to compare the baseline demographic characteristics and various hospital outcomes between AF hospitalizations with and without concomitant RA.

This study has some limitations. Firstly, NIS database studies are subject to all the biases associated with retrospective studies. Secondly, the NIS uses ICD-10 codes to characterize diagnoses and hospitalization events, and hence there is a possibility of errors associated with coding. Thirdly, most of the ICD-10 billing codes used by the database fail to grade disease severity. Thus, we could not determine if underlying disease severity affected the outcome of AF patients with RA. Fourthly, this report reflects data on AF hospitalizations rather than on individual patients, and, therefore, individuals hospitalized multiple times with the same principal diagnosis would be counted multiple times. Finally, data on medications used during hospitalization, adherence to immunosuppressants such as disease-modifying anti-rheumatic drugs (DMARDs) and biologic agents, and laboratory results, which could indicate underlying disease severity and inflammatory activity, are not available in the NIS database.

## Conclusions

Based on our findings, patients admitted primarily for AF with a secondary diagnosis of RA had lesser adjusted mean LOS and total hospital charges compared to those without RA. However, inpatient mortality and the likelihood of undergoing ablation and pharmacologic cardioversion were similar between both groups.

## Appendices

**Table 4:** Supplementary table containing used ICD-10 codes AF: atrial fibrillation; RA: rheumatoid arthritis; MI: myocardial infarction; PCI: percutaneous coronary intervention; CABG: coronary artery bypass graft; COPD: chronic obstructive pulmonary disease; DM: diabetes mellitus; CHF: chronic congestive heart failure; CKD: chronic kidney disease; O_2_: oxygen; AICD: presence of automated implantable cardioverter defibrillator; ICD-10: International Classification of Diseases, Tenth Revision

	ICD-10 codes
Diagnosis codes	
AF	148.0, 148.1, 148.2, 148.91
RA	M05, M06
Procedure codes	
Ablation	258
Pharmacologic cardioversion	3E030RZ, 3E033RZ, 3E040RZ, 3E043RZ, 3E050RZ, 3E053RZ, 3E060RZ, 3E063RZ
Comorbidities	
Dyslipidemia	E78
Old MI	I252
Old PCI	Z9861
Old CABG	Z951
Old pacemaker	Z950
AICD	Z95810
Chronic obstructive pulmonary disease	J41, J42, J43, J44
Carotid artery disease	I652
Old stroke	I63
Hypertension	I10
Peripheral vascular disease	I739
Hypothyroidism	E03
Diabetes mellitus type 1 and 2	E10, E11
Obesity	E660, E6601, E6609, E661, E662, E668, E669
Congestive heart failure	I50
Chronic kidney disease	N18
Liver disease	K70, K71, K72, K73, K74, K75, K76, K77
Electrolyte derangement	E870, E871, E872, E873, E874, E875, E876
Maintenance dialysis	Z992
O_2_ dependence	Z9981
Smoking	Z87891, F17200
Anemia	D50, D51, D52, D53, D55, D56, D57, D58, D59, D60, D61, D62, D63, D64
